# Overestimation of Internal Consistency by Coefficient Omega in Data Giving Rise to a Centroid-Like Factor Solution

**DOI:** 10.1177/00131644241313447

**Published:** 2025-02-13

**Authors:** Karl Schweizer, Tengfei Wang, Xuezhu Ren

**Affiliations:** 1 Goethe University Frankfurt, Germany; 2 Zhejiang University, Hangzhou, China; 3 Huazhong University of Science and Technology, Wuhan, China

**Keywords:** Omega, coefficient Omega, internal consistency, reliability, confirmatory factor analysis

## Abstract

Coefficient Omega measuring internal consistency is investigated for its deviations from expected outcomes when applied to correlational patterns that produce variable-focused factor solutions in confirmatory factor analysis. In these solutions, the factor loadings on the factor of the one-factor measurement model closely correspond to the correlations of one manifest variable with the other manifest variables, as is in centroid solutions. It is demonstrated that in such a situation, a heterogeneous correlational pattern leads to an Omega estimate larger than those for similarly heterogeneous and uniform patterns. A simulation study reveals that these deviations are restricted to datasets including small numbers of manifest variables and that the degree of heterogeneity determines the degree of deviation. We propose a method for identifying variable-focused factor solutions and how to deal with deviations.

## Introduction

This case addresses a peculiarity of coefficient Omega (McDonald, 1999), widely used to evaluate the psychometric quality of multi-item scales for assessing psychological attributes. Coefficient Omega, not only a measure of internal consistency but also considered with respect to congeneric reliability (Lucke, 2005), is derived from confirmatory factor analysis (CFA) according to the congeneric model of measurement (Jöreskog, 1971). Measures of internal consistency are expected to inform the researcher about how closely the items of a scale are interrelated in representing the same construct (Schmitt, 1996; Vogt & Johnson, 2015). Furthermore, special emphasis is given to “the homogeneity among the items” (American Psychological Association [APA], 2018). Although internal consistency is related to reliability, there is no exact correspondence (Lucke, 2005) because reliability is defined as the ratio of variances (Raykov & Marcoulides, 2010, 2019); that describes internal consistency inaccurately. The mentioned peculiarity is regarding correlation patterns leading to a specific factor solution giving rise to an overly large Omega estimate. We refer to it as the *effect of the variable-focused factor solution*. As these correlation patterns display a degree of heterogeneity that contradicts the large Omega estimates, an investigation is conducted that aims at providing additional information on this characteristic and reveals the reasons for the unexpected outcomes, so that correction is possible.

Internal consistency is preferably but not exclusively measured by coefficient Alpha (Cronbach, 1951; Cronbach & Shavelson, 2004) and coefficient Omega (McDonald, 1999). Checks of the quality of these coefficients were performed in taking different perspectives, such as the performance under deviation from tau-equivalence (Gu et al., 2013; Raykov, 1997), under deviation from the homogeneity of the item set (Raykov, 1998; Raykov & Marcoulides, 2015), under deviation from completeness of data (i.e., missing data) (Enders, 2004; Zhang & Yuan, 2016), in data with correlated errors (Gu et al., 2013; Raykov, 2001), in data with non-normality/bias (Bell et al., 2024; Sheng & Sheng, 2012; Xiao & Hau, 2023), in data with sampling error (Deng & Chan, 2017), and in data with outlying observations (Liu et al., 2010; Zhang & Yuan, 2016). Other evaluation studies compared coefficient Alpha with coefficient Omega (e.g., Deng & Chan, 2017; Doval et al., 2023; Hayes & Couts, 2020; Malkewitz et al., 2023; Maydeu-Olivares et al., 2007) and outlined the benefits for application (Raykov, 2024). Furthermore, efficiency-enhancing modifications were proposed (Zhang & Yuan, 2016) and applicability for multivariate measurement was established (Alonso et al., 2010; Tarkkonen & Vehkalati, 2005).

Coefficient Omega is rooted in classical test theory and its modern version, the congeneric test theory proposed by McDonald (1999). Its computation has to be preceded by CFA specified as a one-factor congeneric model of measurement (Brown, 2015; Jöreskog, 1971). CFA aims to check whether the one-factor model accounts for the common systematic variation of data. Also CFA enables obtaining parameter estimates that when appropriately assembled can serve as the analog of the true-score variance. Such true-score variance as numerator of a ratio together with the observed score variance as denominator creates a coefficient that is considered as a measure of reliability defined as ratio of variances (Raykov & Marcoulides, 2010, 2019).

Previous studies have revealed the dependency on tau-equivalence as a characteristic that distinguishes coefficient Alpha from coefficient Omega. Coefficient Alpha is considered as especially sensitive to deviation from tau-equivalence since it implicitly assumes such equivalence (Raykov, 1997). In contrast, congeneric factor loadings allowing for tau-variability instead of enforcing equivalence characterize the input to the computation of coefficient Omega. Instead of sticking to tau-equivalence, this coefficient takes the differences between the items of a scale into consideration and this way overcomes what is considered a disadvantage of coefficient Alpha. As a consequence, restriction because of tau-equivalence is not considered as a problem of coefficient Omega (Deng & Chan, 2017).

**Figure 1.**
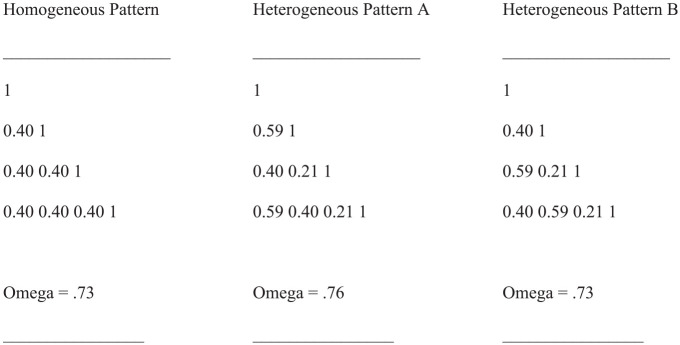
Examples of Correlational Patterns With Equal-Sized Mean Correlations Whereof a Heterogeneous Pattern Yields the Largest Omega Estimate.

Allowing for tau-variability due to congeneric factor loadings opens up the possibility that special correlational patterns of input to CFA exert a special influence on Omega estimates. To demonstrate such special influence of special patterns, we present Figure 1. It includes three lower-triangle matrices composed of correlations plus the corresponding main diagonals. We refer to them as Homogeneous Pattern, Heterogeneous Pattern A and Heterogeneous Pattern B in corresponding order. They share the same mean correlation while two of them even include the same numbers as elements.

As is apparent from the information provided below the patterns, the Omega estimates for Homogeneous Pattern and Heterogeneous Pattern B are identical, whereas Heterogeneous Pattern A yields the largest Omega estimate. These results do not correspond to expectations: first, tau-equivalence suggests that the largest estimate should be observed for Homogeneous Pattern. Second, since Heterogeneous Pattern A and Heterogeneous Pattern B contain identical numerical elements, one would expect their Omega estimates correspond as well.

The investigation of all correlation patterns was conducted using the maximum likelihood estimation method (Jöreskog, 1969) on the basis of the one-factor congeneric measurement model (Brown, 2015). The investigation of Heterogeneous Pattern A led to the largest standardized factor loading (.98, first variable) and the largest range of factor loadings (.57), and of Heterogeneous Pattern B to the second largest factor loading (.87) and the second largest range (.37). In contrast, Homogeneous Pattern yielded same-sized factor loadings (.63) and zero range. The CFA outcomes are referred to as the strong variable-focused factor solution, weak variable-focused factor solution and variables-engaging factor solution, respectively.

The factor loadings observed for Heterogeneous Pattern A closely correspond with the correlations of the first manifest variable of Pattern A with the other manifest variables of Pattern A (λ_1_-r_11_: .98–1.00, λ_2_-r_21_: .60–.59, λ_3_-r_13_: .41–.40, λ_4_-r_14_: .60–.59). In no case, the difference between factor loading and correlation is larger than 0.02. Apparently, in Heterogeneous Pattern A, the first variable is at the *center* of the set of variables. Since the sum of absolute factor loadings is larger for the factor of Heterogeneous Pattern A (2.59) than for Heterogeneous Pattern B (2.50) and for Homogeneous Pattern (2.52), it is reasonable to assume that the factor of Heterogeneous Pattern A is similar to a centroid, as can be determined by the centroid method of factor analysis (Choulakian, 2003; Thurstone, 1935).

What further complicates the situation is that a variable-focused factor solution can be achieved by parameter fixation, such as by scaling the variance parameter according to the marker-variable method (Klopp & Klößner, 2020; Little et al., 2006; Schweizer et al., 2019). This method requires setting one of the factor loadings equal to one in CFA while allowing the variance parameter to be freely estimated. For example, when calculating coefficient Omega for Heterogeneous Pattern B and setting the first factor loading to 1, the Omega estimate rises from .73 to .78. This means that an artificially induced variable-focused factor solution can make the deviation from expectation even more extreme by producing an even larger Omega estimate.

In the following, we provided a formal description of the situation giving rise to the variable-focused factor solution and report two simulation studies designed to reveal the characteristics of correlation matrices that are likely to lead to deviations from expected Omega estimates. We also propose a way of correcting Omega estimates.

### The Outset

Coefficient Omega (McDonald, 1999) is described on the basis of available factor loadings and residual variances. Let *λ*_1_, *λ*_2_, . . . *λ_p_* ∈ℜ be the set of available factor loadings and *ψ*_1_, *ψ*_2_, . . ., *ψ_p_* ∈ℜ the set of residual variances. Then Omega, *ω* ∈ℜ (Equation 6.20b), is defined as:

(1) $$\omega =\frac{{\left(\sum_{i=1,p}\lambda_{i}\right)}^{2}}{{\left(\sum_{i=1,p}\lambda_{i}\right)}^{2}+\sum_{i=1,p}\psi_{i}}.$$

The factor loadings and residual variances originate from CFA of the available correlation matrix. The congeneric measurement model of CFA (Brown, 2015; Graham, 2006) assumes *p*-centered manifest variables included in *p* × 1 vector **x** (**x** $\in {ℜ}^{p\times 1}$ ), *p*-factor loadings included in *p* × 1 vector **λ** (**λ** $\in {ℜ}^{p\times 1}$ ), latent variable *ξ* (*ξ* ∈ℜ ), and *p* residual variances included in *p* × 1 vector **δ** (**δ** $\in {ℜ}^{p\times 1}$ ). The definition of the centered version of this measurement model is given by:

(2) x=λξ+δ.

The corresponding model of the correlation matrix that is employed for parameter estimation is provided by:

(3) $$\sum =\lambda \;\phi \;\lambda^{\prime }+\Psi_{\delta }$$

where **Σ** (**Σ** $\in {ℜ}^{p\times p}$ ) is the *p* × *p* model-implied covariance matrix, **Ψ**_δ_ (**Ψ** $\in {ℜ}^{p\times p}$ ) is the *p* × *p* diagonal matrix of residual variances, **λ** (**λ** $\in {ℜ}^{p\times 1}$ ) is *p* × 1 vector of factor loadings, and *φ* ( φ∈ℜ ) is the variance parameter. If *φ* is set equal to one, parameter estimation yields the factor loadings and residual variances necessary for computing *ω*.

A version of Equation 1 that better aligns with the notation of the entries of the model-implied covariance matrix is achieved by transforming the sums into the vectors. This is achievable by multiplying the column vector, **λ**, from the left with the *p* × 1 unity vector, **1**, as a row vector, that is,

(4) 1′λ

so that the sum of the elements of **λ** is obtained. Furthermore, computing the trace of $\Psi_{\delta }$ is required:

(5) $$\mathrm{tr}\left(\Psi_{\delta }\right)$$

Given these preparatory transformations (Equations 4 and 5), the version of Equation 1 using matrix notation is obtained by:

(6) $$\omega =\frac{{\left(1\prime \lambda \right)}^{2}}{{\left(1\prime \lambda \right)}^{2}+\mathrm{tr}\left(\Psi_{\delta }\right)}$$

(for a similar equation see, e.g., Lucke (2005, Equation 19); the only difference is that Luke chose to repeat **1’λ** which yields a scalar corresponding to the sum of factor loadings in a transformed way instead of simply computing the square.

### The Effect of Variable-Focused Factor Solutions

The effect of a variable-focused factor solution (VFFS) on the omega coefficient explains the observation of the large Omega estimate reported in the introductory section. It is observed in a situation where the latent variable that accounts for the common systematic variation of data coincides with one of the variables. This variable is referred to as *V*. An example of such a situation is provided by Heterogeneous Pattern A. Given a correlation matrix as input to the factor analysis, in such a case, the factor loading of *V* on the latent variable is 1, while the other factor loadings correspond to the correlations of these variables with variable *V*. Note. Experiences with this type of situation suggest that there is mostly no exact correspondence. Instead, there may be a tiny deviation that disappears after rounding.

The effect on the Omega coefficient is caused through the sum of residual variances [tr(**Ψ**)] (Equation 6) since the square of the sum of factor loadings [(**1’λ**)^2^] appears in the numerator and the denominator. In the case of VFFS, the residual variance of variable *V* is zero so that:

(7) $$\mathrm{tr}(\Psi {)}_{\mathrm{VFFS}}=\mathrm{tr}(\Psi )-\Psi_{V}$$

whereas in other situations, all residual variances count. In *p* variables, there are *p*-residual variances except of in the case of VFFS, so that the number of residual variances is reduced to *p* − 1. A smaller number of residual variances can mean a smaller contribution to the denominator and, as a consequence, a larger Omega estimate. What is already apparent at this point is that the larger the number of variables, the smaller is the effect of VFFS since for *p*→∞, *p−*1 / *p*→ 1.

Next, we demonstrate that the effect of VFFS in a correlation matrix like Heterogeneous Pattern A can lead to an Omega estimate that is larger than the Omega estimate for a uniform correlation matrix referred to as UCM in a more general way. The previous considerations reveal that the largest VFFS effect can be expected in small numbers of variables. Therefore, we demonstrate this effect in a set of four variables, as in the example. We assume four variables that give rise to a pattern similar to that of Heterogeneous Pattern A, that is, we assume a mean correlation, *a* (0 < *a* < 1), increments and decrements of the same size, *g* (0 < *g* < 1), and 1 as upper limit for the sum (0 < *a*+*g* < 1), so that a pattern according to Heterogeneous Pattern A is achieved. In this case, the VFFS factor loadings are 1, *a*+*g*, *a* and *a*+*g* in corresponding order. The corresponding residual variances are 0, 1 − (*a*+*g*)^2^, 1 − *a*^2^, and 1 − (*a*+*g*)^2^. In contrast, the correlations of the uniform correlation matrix have the same size, *a*. The correlations of this matrix are reproduced by factor loadings of *a*^1/2^. Therefore, all residual variances are 1 − *a*.

For finding out whether the Omega estimate associated with the VFFS effect or the estimate based on the uniform correlation matrix is the larger one, the residual variances need to be compared. The smaller one can be expected to yield the larger Omega estimate. Starting with the second and fourth residual variances, 2 × [1 − (*a* + *g*)^2^]_VFFS_ and 2 × (1 − *a*)_UCM_ need to be compared. In this case, we select specific g* from the set of possible *g*s such that (*a* + g*)^2^ = *a*. If *g* would be even larger, this would mean a larger Omega–_VFFS_. However, equality allows us to omit these residual variances from the comparison and to concentrate on the remaining residual variances that are now considered as sums: [0 + (1 − *a*^2^)]_VFFS_ and [2 × (1 − *a*)]_UCM_. Since (1 − *a*^2^) = (1 + *a*) × (1 − *a*), both sums can be divided by (1 − *a*) that leaves (1 + *a*) _VFFS_ and 2_UCM_. Because of the assumption of *a* < 1, the following relationship is reached: (1+*a*)_VFFS_ < 2_UCM_ meaning that:

(8) $$\omega_{\mathrm{VFFS}}>\omega_{\mathrm{UCM}}$$

that has to be demonstrated. Note. The selection of *g* larger than g* should even increase the difference between the two Omega estimates (for more details, see Appendix).

### Identifying Variable-Focused Factor Solution

The key role in determining coefficient Omega is played by **λ** that is data-driven. It can reflect the characteristics of the variables-engaging factor solution, of the variable-focused factor solution and of all factor solutions along the continuum between these two types. A uniformity pattern of the correlation coefficients gives rise to the variables-engaging factor solution that is characterized by:

(9) $$\lambda_{1}=\lambda_{2}=\ldots =\lambda_{p}$$

where *λ* is a factor loading and *p* gives the number of manifest variables. In contrast, in the extreme case of a perfect variable-focused factor solution, the factor loadings correspond to the correlations of the variable which represents the center of the set of variables, *x_i_*, with the other variables:

(10) $$\lambda_{1}-{r_{i}}_{1}=\lambda_{2}-{r_{i}}_{2}=\ldots =\lambda_{p}-r_{\mathrm{ip}}=0$$

where *λ* is a factor loading, *r* is a correlation, and *p* gives the number of manifest variables.

Identifying a case of a variable-focused factor solution requires computing the difference between the vector of factor loadings, **λ**, and the selected column vector taken from the correlation matrix, **r**_*i*_, where *i* identifies the column of the correlation matrix that is in the focus of the variable-focused factor solution. The differences are then squared and summed. This means that the coefficient for identifying variable-focused factor solutions, *ν* ∈ℜ , is given by:

(11) $$v=(\lambda -r_{i})\prime \times (\lambda -r_{i}).$$

The smaller the difference between the variable-focused factor and the vector of correlations, the smaller is *ν*. In the case of perfect correspondence, *ν* = 0, as is suggested by Equation 10.

In contrast, in the case of a perfect variables-engaging factor solution typically observed when investigating a uniform matrix (except of the main diagonal), the differences between individual factor loadings and their corresponding correlations are all but one the same since the relationships between factor loadings and correlations are all but one the same (also see Equation 9): *λ* = *r*^1/2^. This means for *ν*_uniform_ that:

(12) $$v_{\mathrm{uniform}}=\left(p-1\right)\times {\left(r^{1/2}-r\right)}^{2}+{\left(r^{1/2}-1\right)}^{2}$$

where *p* gives the number of manifest variables. The exception is regarding the number taken from the main diagonal of the correlation matrix. Except of for *r* = 1, *ν*_uniform_ > 0. Coefficient *ν* for Homogeneous Pattern (Equation 9) is 0.2975, for Heterogeneous Pattern A 0.0007, for Heterogeneous Pattern B 0.0385.

### A Note Regarding the Prevalence of Factor-Focused Factor Solutions

**Figure 2.**
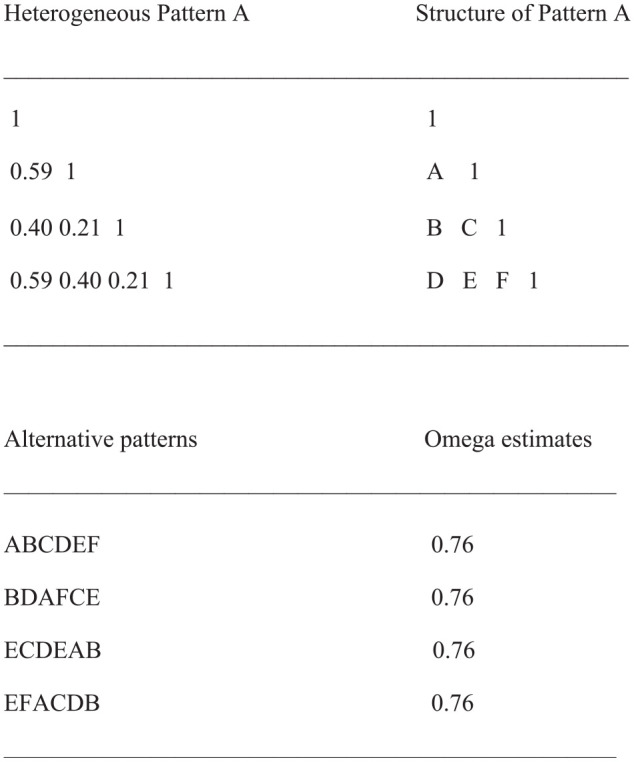
Permutations of Coefficients of Heterogeneous Pattern A Leading to Variable-Focused Factor Solutions and Overly Large Omega Estimates Out of a Sample of 10 Random Takes.

To find out whether Homogeneous Pattern A is a rare example leading to a variable-focused factor solution or whether there are other arrangements of the numbers displaying similar properties, we generated 10 random permutations. The results revealed four patterns, all including the same numbers, which also gave rise to a variable-focused factor solution and an Omega estimate of 0.76. Figure 2 presents these patterns.

All patterns include the same numbers identified by the same letters. This analysis shows that Heterogeneous Pattern A is not unique but a member of a group of patterns sharing the same properties.

## The Simulation Studies

The general objective of the simulation studies (design study and generalization study) was to investigate how deviation from uniformity of the input to CFA, indicating heterogeneity, influenced the Omega estimate, while keeping a constant average correlation. The structure giving rise to a variable-focused factor solution was selected for creating deviation, that is, heterogeneity. The *design study* started from a uniform correlation matrix with the exception of the main diagonal and increased the degree of heterogeneity among the elements of the correlation matrix that was input to CFA while the effect on coefficient Omega was investigated. Other design factors characterized the *generalization study.* They were variation in the number of variables, that is, the size of a correlation matrix, and in the level of the average correlation.

### Method

The method section in large parts applies to both studies as there was a considerable overlap regarding methods. A major issue was the development of an appropriate simulation method since the standard method (Jöreskog & Sörbom, 2001) proved to be unfit for generating simulated data expected to display various degrees of heterogeneity when the number of columns/rows of a matrix was large. This standard method consisted in generating random data on the basis of a relational pattern and then transforming them into covariance matrices. Proceeding in this way, the heterogeneity, as was characteristic of Heterogeneous Pattern A (see Figure 1), did no more characterize covariance matrices in larger numbers of columns/rows. Instead there was basic uniformity with random deviations.

The final method consisted in the generation of normally distributed random data [*N*(0,1)] using program package PRELIS (Jöreskog & Sörbom, 2001). These data were then combined with the relational pattern in a systematic way, so that new patterns resulted that randomly deviated from the original relational pattern. In this way, 300 modified relational patterns were made available for serving as correlation matrices that were input to CFA for a structural investigation.

The generation of the elements of a modified relational pattern, *a*_new_, started from the elements of the original relational pattern, *a*_old_. Element *a*_old_ was combined with the standardized and weighted (1/10) random number, *c*, that could be positive or negative to give the new number, *a*_new_, in the following way:

$$a_{\mathrm{new}}=a_{\mathrm{old}}\times \left(1+c/10\right).$$

Because of the mean of zero of the set of generated random numbers, an individual random number was either positive or negative, so that finally, there was either an unpredictable small increase or decrease that distinguished *a*_new_ from *a*_old_.

Furthermore, we conceptualized the patterns as composed of triplet sequences including a large number, a mean-sized number, and a small number (e.g., 0.60, 0.40, 0.20) in the case of a heterogeneous pattern, and numbers of the same size (e.g., 0.40, 0.40, 0.40) in the case of a corresponding homogeneous pattern. If the triple sequence did not fit to the number of entries of a row, it was continued in the next row or replaced by the mean of the sequence. For example, the patterns of Figure 1 included two triplets; the first triplet extended to rows two and three and the second triplet was included in the fourth row (the numbers of the main diagonal ignored).

#### Degree Study

When investigating the effect of the degree of heterogeneity of the elements of the correlation matrix, the following triplet sequence provided the outset: 0.40, 0.40, and 0.40. Heterogeneity was created by increasing the first number and decreasing the third number of the triple sequence. This was achieved by multiplication with a specific probability. For example, using a probability of .50 for the 0.4-mean level would lead to a high number of 0.60 (0.4 + 0.2) and a small number of 0.20 (0.4 − 0.2) so that the consequential triple sequence included 0.60, 0.40, and 0.20. The considered probability coefficients were .10, .20, .30, .40, .50, and .60. The outcomes of the multiplication served as input, *a*_old_, to the random manipulation leading to *a*_new_. A homogeneous pattern was also considered but there was no multiplication with probability coefficients.

#### Generalization Study

When investigating the effects of the number of variables and the average level of correlations, additional triplet sequences had to be added to the correlational pattern serving as relational patterns. Starting from the examples that were correlational patterns including four columns/rows, new patterns including 5, 6, 9, 12, and 20 columns/rows were constructed by adding triplet sequences. To check the influence of the mean level of the correlations, correlational patterns were created with mean sizes of 0.2, 0.3 besides 0.4. Larger mean sizes were omitted because they were found to lead to correlation matrices that were not positive-definite in combination with larger numbers of columns/rows.

The software package LISREL (Jöreskog & Sörbom, 2006) was employed using the maximum likelihood estimation option for investigating the simulated data. The measurement model for CFA was specified as a one-factor congeneric model (Equation 2). We employed the following fit indices and established criteria (in parentheses) for the evaluation of the results of this study: χ^2^, root mean square error of approximation (RMSEA; ≤ 0.06), Standardized Root Mean Square Residual (SRMR; ≤ 0.08), Nonnormed Fit Index (NNFI; ≥ 0.95), and comparative fit index (CFI; ≥ 0.95) (see DiStefano, 2016; Schweizer, 2010). A TURBO PASCAL program was used for extracting factor loadings and residual variances from LISREL output and the computation of McDonald’s Omega (Equations 1 and 6).

### Results

#### Degree Study: Results for Different Degrees of Heterogeneity

**Figure 3.**
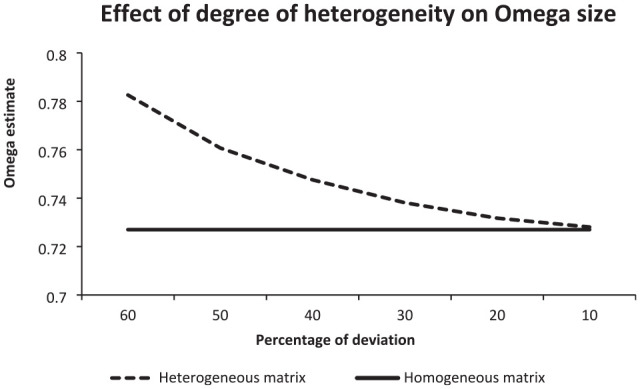
Illustration of the Mean Omega Estimates Observed for Heterogeneous Correlation Matrices That Displayed Different Percentages of Deviation (= Degrees of Heterogeneity) and Also for (Constant) Homogeneous Matrices.

The investigation of the effect of the different degrees of heterogeneity on Omega was conducted using 4 × 4 correlation matrices with a mean size of correlation coefficients of 0.40. Since the probability coefficients of .10, .20, .30, .40, .50, and .60 led to deviations of 10%, 20%, 30%, 40%, 50%, and 60% from 0.40 in both directions, we refer to them as percentages. In each percentage, the number of simulated matrices serving as input to CFA was 300. Figure 3 illustrates the results.

The curve illustrating the course of the mean Omega estimates observed for heterogeneous correlation matrices displayed a nonlinear decrease from 60% to 10%. The decrease was steeper in the beginning and less steep in the end (from the left to the right). In contrast, the curve representing the mean Omega estimates observed for homogeneous correlation coefficients kept a constant distance to the horizontal axis as there was no probability manipulation, that is, the same estimate was used for all percentages.

In the deviation of 60%, the mean Omega was 0.782 (*SD* = 0.023) for heterogeneous matrices and 0.727 (*SD* = 0.016) otherwise, while in the deviation of 10%, it was 0.728 (*SD* = 0.016) for heterogeneous matrices and 0.727(*SD* = 0.016) for homogeneous matrices. The mean difference was 0.055 for 60%; it decreased to 0.001 for 10%.

#### Generalization Study: Results for Different Numbers and Mean Coefficient Sizes

**Figure 4.**
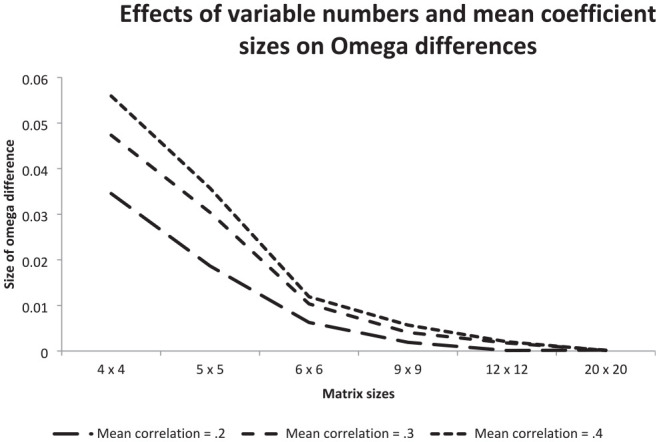
Illustration of the Effects of the Number of Columns/Rows of Matrices Input to CFA and the Size of the Mean Correlation on the Difference Between the Omegas for Heterogeneous and Homogeneous Matrices.

The investigation of the effects of the number of columns/rows of the matrices that were input to CFA and of the mean sizes of the coefficients included in the matrices on the mean Omega estimates provided the basis of the results reported in Figure 4.

The curves of Figures 4 represent differences between mean Omega estimates obtained for heterogeneous matrices and corresponding homogeneous matrices. Each curve reflects another mean size of coefficients. All curves decreased from the left-hand side to the right-hand side of Figure 4. This indicated that the larger the number of columns/rows was, the smaller the Omega difference between homogeneous and heterogeneous matrices. The steepness was largest in the range of matrices with four to six columns/rows.

Furthermore, the differences between the curves included in Figure 4 demonstrated that the mean size of the coefficients counted. The mean size of 0.4 led to the largest Omega difference and the mean size of 0.2 to the smallest Omega difference. The Omega differences were largest in 4 × 4 matrices: in the mean size of 0.4, it was 0.055, in the mean size of 0.3, it was 0.048, and in the mean size of 0.2, it was 0.034. In 20 × 20 matrices, the differences for all mean sizes were virtually zero.

In sum, in almost all cases, heterogeneity according to the pattern leading to the variable-focused factor solution increased Omega. The larger the heterogeneity, the larger the estimate was. Furthermore, the mean size of the correlations and the number of row/columns played a role. Although increasing the mean size increased the effect on Omega, the increase in the number of rows/columns decreased it.

## A Proposal for an Adjustment Method

An adjustment method is proposed that is based on the observation that the Omega estimates for heterogeneous correlation matrices, **R**_heterogeneous_, approach the Omega estimates for the corresponding homogeneous correlation matrices, **R**_homogeneous_, in larger numbers of columns/rows of correlation matrices and on the assumption that equal relationships among variables should lead to equal Omegas. This observation suggests the replacement of the Omega for the heterogeneous correlation matrix, *ω*_heterogeneous_, by the Omega for the corresponding homogeneous matrix that is now referred to as *ω*_adjusted_. This replacement is achievable by substituting the off-diagonal elements of **R**_heterogeneous_, *r_ij_* (*i*, *j* = 1, . . ., *p* and *i*≠*j*), by their mean which we refer to as adjusted correlation, *r*_adjusted_*ij*_:

$$r_{\mathrm{adjusted}\_\mathrm{ij}}=\bar{r}$$

where $\bar{r}$ is the mean of the off-diagonal elements of **R**_heterogeneous_. The matrix with the replaced elements, **R**_adjusted_, provides the input to CFA for the achieving factor loadings and the computation of *ω*_adjusted_ according to Equation 6. However, this replacement is only recommended for cases where the coefficient for identifying variable-focused factor solutions, *ν* is close to zero.

## Discussion

Coefficient Omega displays an unexpected degree of sensitivity for types of patterns of relationships among a set of variables. The results suggest that there are at least two types: the type of patterns that give rise to a variable-focused factor solution and the type of patterns leading to a variables-engaging factor solution. Evidence of these types is available from the various Omega estimates observed in investigating the examples and the simulation studies. This sensitivity impairs Omega’s role as indicator of internal consistency (Schmitt, 1996; Vogt & Johnson, 2015). In small numbers of variables, different estimates are achievable instead of equal estimates. The same set of correlations among variables leads to estimates that can vary within a range including correct estimates besides overly large estimates.

Overestimation of internal consistency has also been reported by Gu et al. (2013) who observed it because of correlated errors while Raykov (2001) found bias due to correlated error. Correlated errors are usually due to an especially large degree of similarity between pairs of variables included in a scale (Schweizer et al., 2023, 2024). The variables of such a pair have more in common than what the remaining variables have in common. Because of the large size of such a correlation between the variables of such a pair, it usually contributes to the heterogeneity among variables. Furthermore, there is research on the effect of outliers on internal consistency. Outliers can mean skewed and extreme scores with consequences for correlations. Outliers can (but must not) lead to particularly large but also to smaller than otherwise expected correlations. Both types of effects contribute to the heterogeneity of a pattern of correlations. However, outliers are reported to be mainly associated with a decrease in internal consistency instead of an increase when assessed by coefficient Omega (Zhang & Yuan, 2016).

Although there appears to be support for the reported results, it needs to be pointed out that the comparability of the studies is limited. Although the idea of a correlated error is typically restricted to pairs of variables, the Gu et al.’s study following the study by Raykov assumes subsets of variables with increased correlations among each other. Such subsets may increase the heterogeneity at a general level of structure but not so much in the vicinity of individual variables. Such subsets provide the basis for method factors, as are included in multi-trait–multimethod models (Byrne, 2016). The correlations among the variables of a subset of variables may be larger than the correlations with variables of other subsets but are likely to be homogeneous within the same subset meaning that there is no increase in the type of heterogeneity, as was the characteristic of the example matrices.

The pattern of factor loadings observed in investigating Heterogeneous Pattern A displays the characteristics of centroids observable when data are investigated by the centroid method of factor analysis (Choulakian, 2003; Thurstone, 1935). In the example matrices, the similarity to a centroid is not only obvious from the factor loadings but also from the factor variances: the scaled factor variance (Schweizer et al., 2019) for Heterogeneous Pattern A is 1.85, for Heterogeneous Pattern B 1.65, and for Homogeneous Pattern 1.59. This finding suggests that under special conditions, even modern CFA can yield factor solutions similar to factor solutions obtainable by the centroid method that is nowadays only considered in investigations in the framework of the Q methodology (Ramlo, 2015).

Variable-focused factor solutions leading to overly large Omega estimates are only reported for rather small numbers of variables, suggesting that a small number is a property that is favorable for this kind of factor solutions. This observation gains special importance in the model-fit approach (Gumedze & Dunne, 2011; Jöreskog, 1970) that consists in the reproduction of the empirical covariance matrix by the model-implied matrix that are square matrices. A characteristic of such a matrix is a dynamic relationship between the number of variables (= columns/rows) and the number of off-diagonal elements of the lower-triangle matrix. In small matrices (3, 4 variables), it is an equality or near-equality relationship (1/1,1/1.5 variables/elements), whereas in larger matrices (5, 6 columns/rows), the number of off-diagonal elements is much larger than the number of variables (1/2,1/2.5 variables/elements). As a consequence, in large matrices, a factor loading contributes to the reproduction of more correlations than otherwise and, therefore, is more likely to reproduce average correlations exactly than extreme correlation. The contrary is observable in small numbers of variables.

Finally, the question needs to be addressed whether correction is necessary and how it is to be conducted. Since internal consistency is expected to indicate how closely the items of a scale are related to each other in representing the same construct (Schmitt, 1996; Vogt & Johnson, 2015), the focus is not so much on the integration of the items into a whole but on the relationships among each other. Since the relationships among the items of different permutations are the same, as for example Heterogeneous Pattern A and Heterogeneous Pattern B, internal consistency suggests a correction, so that the outcomes for the permutations are the same. We propose the following: start with identifying such a case using Equation 9. In the case that the outcome is close to zero (< 0.01), transform the heterogeneous matrix into a uniform matrix (with the exception of the main diagonal). Finally, repeat the computation with the new uniform matrix as input. Note. We like to make the reader aware that the definition of reliability as ratio of variances (Raykov & Marcoulides, 2010, 2017) would not require such a correction.

A possible limitation of the study is that we only investigated a subset of all possible permutations of the coefficients of Heterogeneous Pattern A but determining the exact percentage was not an aim of the study and would not have changed the conclusion. Furthermore, there was no systematic search for other special patterns than the patterns including four variables.

In sum, the reported research reveals that Omega estimates vary as a function of the type of the pattern of coefficients in small numbers of variables. The range of variation includes overly large Omega estimates besides correct estimates. Overestimation occurs when CFA yields a variable-focused factor solution as input to the Omega calculation. A method for identifying and adjusting such cases is proposed.

## Appendix

**Table:**

VFFS matrix:		Uniform matrix UCM)	
1		1	
*a*+*g* 1		*a* 1	
*a a−g* 1		*a a* 1	
*a*+*g a a−g* 1		*a a a* 1	
VFFS factor loadings		UCM factor loadings	
Residual variances		Residual variances	
1	0	*a* ^1/2^	(1*−a*)
*a*+*g*	1*−*(*a*+*g*)^2^	*a* ^1/2^	(1*−a*)
*A*	1*−a*^2^	*a* ^1/2^	(1*−a*)
*a*+*g*	1*−*(*a*+*g*)^2^	*a* ^1/2^	(1*−a*)
Modification 1: select *g** such that (*a*+*g**)^2^ = *a* (*a + g* <1)			
Modification 2: (1*−a*^2^) = (1+*a*) (1*−a*)			
Residual variances		Residual variances	
0			(1*−a*)
1*−*(*a*+*g**)^2^		=	(1*−a*)
(1 +*a*) (1*−a*)			(1*−a*)
1*−*(*a*+*g**)^2^		=	(1*−a*)
Modification 3: omission of equality relationships			
Residual variances		Residual variances	
0		(1*−a*)	
(1+*a*) (1*−a*)		(1*−a*)	
Modification 4: summation			
Residual variances		Residual variances	
0 + (1+*a*) (1*−a*)		2 (1*−a*)	
Modification 5: division by (1*−a*)			
Residual variances		Residual variances	
(1+*a*)		2	
Modification 6 (subtraction of 1) and final comparison for *a* < 1 and *g*≥*g**:			
VFFS: residual variances remainder		UCM: residual variances remainder	
*a*		<	1 .
